# Epidemiology of childhood tuberculosis after ceasing universal Bacillus Calmette–Guérin vaccination

**DOI:** 10.1038/s41598-021-95294-y

**Published:** 2021-08-05

**Authors:** Sayori Kobayashi, Takashi Yoshiyama, Kazuhiro Uchimura, Yuko Hamaguchi, Seiya Kato

**Affiliations:** The Research Institute of Tuberculosis, Japan Anti-Tuberculosis Association (RIT/JATA), 3-1-24 Matsuyama, Kiyose, Tokyo, 204-8533 Japan

**Keywords:** Epidemiology, Paediatric research, Tuberculosis, Vaccines

## Abstract

Universal Bacillus Calmette–Guérin (BCG) vaccination is recommended in countries with high tuberculosis (TB) burden. Nevertheless, several countries have ceased universal BCG vaccination over the past 40 years, with scarce comparative epidemiological analyses regarding childhood TB after the policy change. We analysed data on childhood TB in countries that ceased universal BCG vaccination. Data sources included national/international databases, published papers, annual TB reports, and public health authority websites. Childhood TB notification rate increased in one of seven countries with available data. Pulmonary TB and TB lymphadenitis were the main causes of increasing childhood cases, while changes in severe forms of TB cases were minor. Maintaining high vaccine coverage for the target group was a common challenge after shifting selective vaccination. In some countries showing no increase in childhood TB after a BCG policy change, the majority of childhood TB cases were patients from abroad or those with overseas parents; these countries had changed immigration policies during the same period. Heterogeneity in childhood TB epidemiology was observed after ceasing universal BCG vaccination; several factors might obscure the influence of vaccination policy change. Lessons learned from these countries may aid in the development of better BCG vaccination strategies.

## Introduction

Tuberculosis (TB) remains a major public health concern, with an estimated 10 million cases globally in 2018^1^. The Bacillus Calmette–Guérin (BCG) vaccine is currently the only effective vaccine against TB, and universal BCG vaccination, i.e. vaccination of all individuals up to a certain age, is widely conducted in TB high burden countries (HBCs).

Considering the risk–benefit balance (i.e., the balance between the benefit and risk of administering the BCG vaccine in vaccinated populations) after decreasing the TB burden, TB low or middle burden countries have terminated universal BCG vaccination. In 1994, the International Union Against Tuberculosis and Lung Disease (IUATLD) recommended using three indicators to decide the discontinuation of universal BCG vaccination: an average annual notification rate of sputum smear-positive pulmonary TB of ≦ 5 per 100,000 population over the previous 3 years; an average annual notification rate of TB meningitis in children (aged under 5 years) of < 1 per 10 million general population over the previous 5 years; and an average annual risk of tuberculosis infection of ≦ 0.1%^2^. An increase in the number of childhood TB cases after ceasing universal BCG vaccination is assumed to be a negative impact of the policy change.

Several countries have ceased universal BCG vaccination over the past 40 years, and the epidemiological trends of childhood TB after vaccination policy changes in these countries have rarely been compared. Learning from the experience of these countries is important to understand the effects of the vaccination policy change on childhood TB. Thus, the study aimed to compare the trend in childhood TB notification around the year of cessation of universal BCG vaccination, focusing on TB disease types and the patient’s birthplace, and to identify common challenges.

## Methods

### Inclusion criteria and indicators of interest

Countries that ceased universal BCG vaccination were included in this study. The primary indicator of interest was the short-term change in childhood TB notification by age group (0–4 and 5–14 years old), and the secondary indicators were the changes in childhood TB notification by four disease types and birthplace. Vaccine coverage for the target group after cessation was included as an additional indicator if the data were available.

### Data collection

Based on the World BCG Atlas, 17 countries have ceased universal BCG vaccination (Australia, Austria, the Czech Republic, Denmark, Ecuador, Finland, France, Germany, Israel, New Zealand, Norway, Slovakia, Slovenia, Spain, Sweden, Switzerland, and the United Kingdom [UK])^3^. After excluding Ecuador due to data uncertainty (e.g., other documents have indicated that the country continues universal BCG vaccination), we sent an email to the TB focal bodies of these countries (Ministry of Health [MOH], National Institute of Public Health, etc.) to collect information on notifications of childhood TB by age group, the four types of TB disease (pulmonary TB, TB lymphadenitis, meningeal TB, and miliary TB), patient birthplace, and vaccine coverage.

We obtained relevant data from the Czech Republic, France, Germany, Norway, Slovakia, Switzerland, and the UK. To collect additional data, we searched published academic reports using PubMed and Google, annual TB reports and official documents from the websites of the respective MOHs and national institutes of public health, and the databases of the European Centre for Disease Prevention and Control (ECDC) and the World Bank (Table 1)^4,5^.

**Table 1:** Sources of data.

		Focal bodies	Public database^1^	TB annual report	Published articles
Notification at time of vaccination cessation	The Czech Republic, Germany, Switzerland	✓			
	Finland, France, Norway, Slovakia, Slovenia, the UK		✓		
	Australia, New Zealand			✓	
	Sweden				✓^2^
Annual notification	The Czech Republic, Finland, France, Norway, Slovakia, Slovenia, the UK		✓		
	Germany	✓	✓		
	Switzerland	✓			
Birthplace	France, Norway, the UK	✓			
Disease types	France, Norway, Slovakia, the UK	✓			
Vaccine coverage	The Czech Republic	✓			
	France	✓			✓^3^
	Norway, Slovenia, Sweden				✓^2,4,5^
	The UK			✓	

^1^Surveillance and disease data. European Centre for Disease Prevention and Control (https://www.ecdc.europa.eu/en/surveillance-and-disease-data).

^2^Romanus, V., Svensson, A. & Hallander, H. O. The impact of changing BCG coverage on tuberculosis incidence in Swedish-born children between 1969 and 1989. *Tuber. Lung Dis.* **73**, 150–161 (1992) (Sweden).

^3^Guthmann, J. P., Antoine, D., Fonteneau, L., Che, D. & Lévy-Bruhl, D. Assessing BCG vaccination coverage and incidence of paediatric tuberculosis following two major changes in BCG vaccination policy in France. *Euro. Surveill.* **16**, 19,824 (2011) (France).

^4^Evaluering og revisjon av råd om BCG-vaksinasjon gjeldende fra 1. juni 2018 [Evaluation and revision of advice on BCG vaccination valid from 1 June 2018]. *Folkehelseinstituttet [Norwegian National Institute of Public Health]* (2018). Feiring, B. et al. Do selective immunisation against tuberculosis and hepatitis B reach the targeted populations? A nationwide register-based study evaluating the recommendations in the Norwegian Childhood Immunisation Programme. *Vaccine.* **34**, 2015–2020 (2016) (Norway).

^5^Infoso, A. & Falzon, D. European Survey of BCG Vaccination Policies and Surveillance in Children, 2005. Euro Surveill. **11**, 6–11 (2006) (Slovenia).

### Statistical analysis

Childhood TB notification rates per 100,000 population by disease type (pulmonary TB, TB lymphadenitis, meningeal TB, and miliary TB) in France, Norway, Slovakia, and the UK were calculated from the number of TB patients (aged 0–14 years) categorised by these four disease types (data from Santé Publique France, The Norwegian Institute of Public Health, National Institute for TB, Lung Diseases and Thoracic Surgery in Slovakia, and Public Health England) and population data (individuals aged 0–14 years) from the World Bank. To analyse TB notification rates by these four disease types, case reports without detailed information (i.e., those that only mention extrapulmonary TB) were excluded. STATA (StataCorp. 2019. *Stata Statistical Software: Release 16*. College Station, TX: StataCorp LLC.) was used to calculate the notification rate ratio and 95% confidence interval (CI).

### Ethical approval and consent to participate

Ethical approval was waived and informed consent was not required as no human participants were included in this study (Dr. Masaki Ota, Vice-chairman of The Institutional Review Board, The Research Institute of Tuberculosis, Japan Anti-Tuberculosis Association. Approval number: RIT/IRB 2020-22).

## Results

### Trends in TB notification rate per 100,000 population

On the year of cessation of universal BCG vaccination, TB notification rates per 100,000 population (all forms and all ages) were between 5.5 (Finland, ceased in 2006) and 19.9 (Sweden, ceased in 1975) in the 12 countries for which data were available. The childhood TB notification rate per 100,000 population (0–4 years of age) showed a narrower range, from 0.0 (Finland, ceased in 2006) to 4.7 (the UK, ceased in 2005) among eight countries, excluding Germany and Sweden due to the different age distributions of their accessible data (Table 2, partial data obtained from the Institute of Health Information and Statistics of the Czech Republic and the Swiss Federal Office of Public Health)^4,6–9^.

**Table 2:** TB notification rate (all ages, 0–4 years) at cessation of universal BCG vaccination^1^.

Country	Year of cessation	TB notification rate^2^ (all forms, all ages)	Country	Year of cessation	TB notification rate^2^ (all forms, 0–4 years old)
Finland	2006	5.5	Finland	2006	0.0
Australia^3^	Mid-1980s	5.7	The Czech Republic	2010	0.2
Slovakia	2012	6.4	Sweden^3,5^	1975	0.8 (0–14 years)
The Czech Republic	2010	6.5	Norway	2009	1.3
Norway	2009	7.5	Slovakia	2012	1.4
France	2007	8.8	Switzerland	1987	1.6
New Zealand^3^	1996	11.7	Slovenia	2005	2.2
The UK	2005	13.8	France	2007	3.5
Slovenia	2005	13.9	The UK	2005	4.7
Switzerland^4^	1987	16.5	Germany^3,5^	1976 and 1991	5.8 (1–4 years)
Germany^3^	1976 and 1991	16.8			
Sweden^3^	1975	19.9			

^1^Data sources: Institute of Health Information and Statistics of the Czech Republic, the German Federal Health Monitoring System, the Swiss Federal Office of Public Health, European Centre for Disease Prevention and Control. Surveillance Atlas of Infectious Disease, tuberculosis (Finland, France, Norway, Slovakia, Slovenia, the UK) (https://www.ecdc.europa.eu/en/tuberculosis/surveillance/atlas), the TB annual report (Australia and New Zealand), the published paper: Romanus, V., Svensson, A. & Hallander, H. O. The impact of changing BCG coverage on tuberculosis incidence in Swedish-born children between 1969 and 1989. *Tuber. Lung Dis.* **73**, 150–161 (1992) (Sweden).

^2^Rate per 100,000 population.

^3^The available data are not from the exact year of cessation for the following countries: Australia (1986), Germany (1991), New Zealand (1995), and Sweden (1974).

^4^The year of data is 1988 (2.1 in 1987, which was the first year of data collection).

^5^The data are for patients 1–4 years of age for Germany and 0–14 years of age for Sweden.

*BCG* bacillus Calmette–Guérin, **TB** tuberculosis, *UK* United Kingdom.

**Figure 1:**
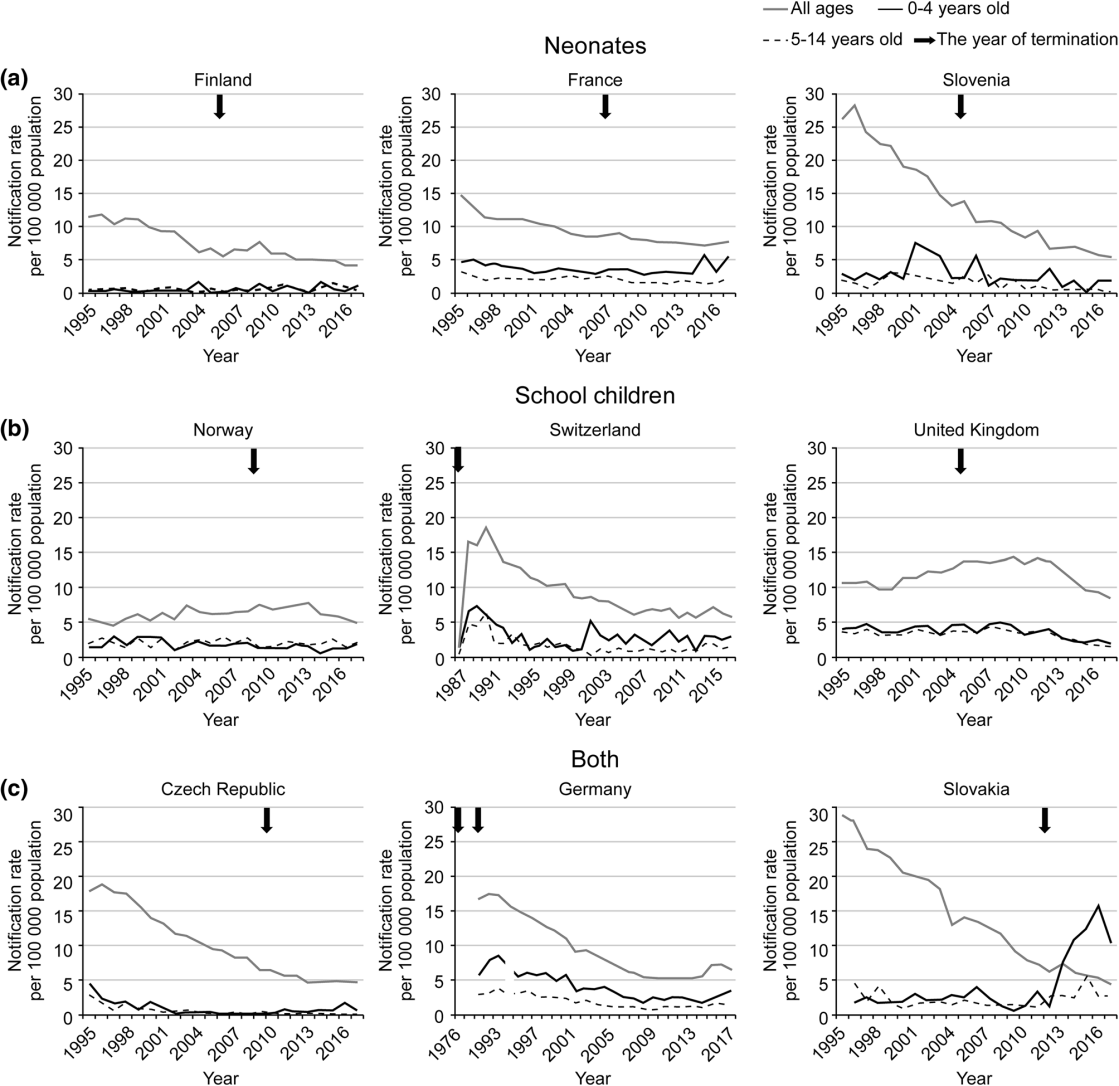
Trends in tuberculosis notification rate (all forms; all ages, 0–4 years, and 5–14 years). *Data sources: ECDC data (the Czech Republic, Finland, France, Germany (1995–2017), Norway, Slovakia, Slovenia and the UK) (https://www.ecdc.europa.eu/en/surveillance-and-disease-data), The Germany Federal Health Monitoring System (1991–1994) (http://www.gbe-bund.de/stichworte/Tuberkulose.html), and the Swiss Federal Office of Public Health. ^†^TB notification rate per 100,000 population. ^‡^Figures are categorised into three groups by the target age of BCG vaccination (a: neonates, b: school children, c: both neonates and school children), and the arrow in figures shows the year of cessation. ^§^The data from 1991 to 1994 in Germany include different age distributions (1–4 and 5–15 years). Abbreviations: Bacillus Calmette–Guérin (BCG); United Kingdom (UK).

The annual TB notification rates per 100,000 population (all forms, 0–4 and 5–14 years of age) before and after vaccine cessation were available for seven countries (the Czech Republic, Finland, France, Norway, Slovakia, Slovenia, and the UK). For two countries (Germany and Switzerland), only data from the year of cessation were available (Fig. 1, partial data obtained from the Swiss Federal Office of Public Health)^4,9^. When comparing the 5-year data before and after cessation among the first group of seven countries, the notification rates increased in both age groups (0–4 and 5–14 years) only in Slovakia; the notification rate ratios (NRR) were 7.1 (95% CI 4.2–13) in the 0–4 years group and 2.0 (95% CI 1.1–3.8) in the 5–14 years group (Table 3)^4^. Although we do not have annual data from Sweden, the study indicated that the TB incidence per 100,000 person-years in unvaccinated children born between 1975 and 1980 was 1.4, which was approximately five times higher than that in vaccinated children born between 1969 and 1974 (0.3)^10^.

**Table 3:** Comparison of pre- and post-cessation period TB notification rate per 100,000 population.

Country	Year		Notification (0–4 years)^1^			Notification (5–14 years)^1^			Notification (0–14 years)^2^			
			All forms			All forms			Meningeal TB		Miliary TB	
	Pre-period	Post-period	Mean (95% CI)		NRR (95% CI)	Mean (95% CI)		NRR (95% CI)	Mean (95% CI)		Mean (95% CI)	
			Pre-period	Post-period		Pre-period	Post-period		Pre-period	Post-period	Pre-period	Post-period
The Czech Republic	2006–2010	2011–2015	0.3 (0.1 to 0.4)	0.7 (0.5 to 0.8)	2.3 (0.5 to 14)	0.3 (0.1 to 0.5)	0.2 (0.1 to 0.2)	0.7 (0.1 to 5.8)	NA			
Finland	2002–2006	2007–2011	0.5 (-0.6 to 1.6)	0.7 (0.7 to 0.8)	1.4 (0.4 to 5.6)	0.4 (-0.3 to 1.2)	0.7 (0.7 to 0.8)	1.8 (0.5 to 8.2)	NA			
France	2003–2007	2008–2012	3.3 (3.0 to 3.7)	3.2 (2.8 to 3.6)	1.0 (0.6 to 1.6)	2.4 (2.2 to 2.6)	1.6 (1.3 to 1.8)	0.7 (0.3 to 1.3)	0.02 (0.01 to 0.03)	0.02 (0.01 to 0.03)	0.003 (-0.01 to 0.01)	0.005 (0.00 to 0.01)
Norway	2005–2009	2010–2014	1.8 (1.4 to 2.2)	1.4 (0.8 to 2.0)	0.8 (0.4 to 1.7)	2.3 (1.7 to 3.0)	1.9 (1.6 to 2.2)	0.8 (0.4 to 1.6)	0	0.02 (-0.04 to 0.08)	0.04 (-0.03 to 0.12)	0.04 (-0.03 to 0.12)
Slovakia	2008–2012	2013–2017	1.7 (0.4 to 3.0)	12 (7.8 to 15)	7.1 (4.2 to 13)	1.7 (1.0 to 2.5)	3.4 (1.8 to 5.0)	2.0 (1.1 to 3.8)	0	0.07 (-0.01 to 0.15)	0	0.14 (0.02 to 0.27)
Slovenia	2001–2005	2006–2010	4.9 (1.8 to 8.0)	2.6 (0.4 to 4.7)	0.5 (0.3 to 0.9)	2.2 (1.6 to 2.8)	1.5 (0.3 to 2.7)	0.7 (0.3 to 1.4)	NA			
The UK	2001–2005	2006–2010	4.2 (3.5 to 5.0)	4.2 (3.2 to 5.1)	1.0 (0.6 to 1.6)	3.7 (3.3 to 4.0)	3.8 (3.3 to 4.3)	1.0 (0.6 to 1.7)	0.10 (0.03 to 0.16)	0.11 (0.06 to 0.15)	0.05 (0.04 to 0.07)	0.06 (0.02 to 0.10)

^1^Data source: ECDC (https://www.ecdc.europa.eu/en/surveillance-and-disease-data).

^2^Data sources: The World Bank for population data; Sante Publique France; The Norwegian Institute of Public Health; National Institute for TB, Lung Diseases and Thoracic Surgery in Slovakia, and Public Health England for the number of TB notifications.

*CI* confidence interval, *NRR* notification rate ratio, *TB* tuberculosis, *UK* United Kingdom.

More detailed data for Slovakia demonstrated that the TB notification rate among patients aged 0–4 years had been increasing since 2013 (one year after cessation), with the peak at 15.7 per 100,000 population in 2016, in contrast to the continuously declining trend in the overall population (Fig. 1). The mean annual number of TB notifications in patients aged less than 15 years was 15 (range: 11–19) from 2007–2012, and 85% of these were from the Roma population. The corresponding value from 2013–2018 increased to 50 (range: 38–67), with a similar proportion from the Roma population (86%), which has been the target of selective vaccination (partial data obtained from the National Institute for TB, Lung Diseases and Thoracic Surgery in Slovakia)^4^. The report indicated that this increase might be owing to a combination of factors such as improvements in the surveillance system and case detection, as well as the vaccination policy change^11^.

### Birthplace-related characteristics among children with TB (0–14 years of age)

Data on childhood TB patients categorised by birthplace before and after cessation were available for three countries: France, Norway, and the UK.

**Figure 2:**
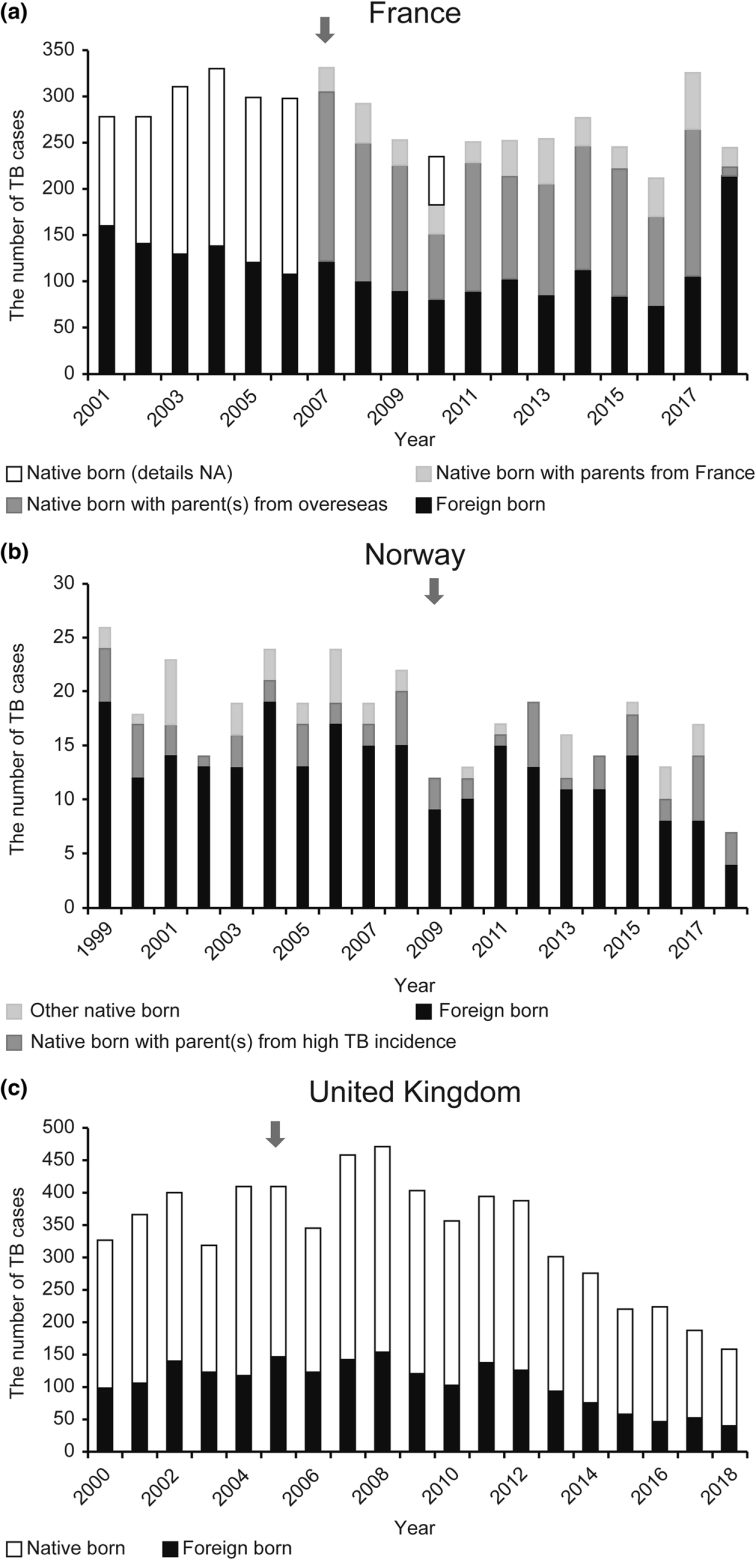
Birthplace-related characteristics among children with tuberculosis (0–14 years of age). *Data sources: Santé Publique France, Norwegian Institute of Public Health, and Public Health England. ^†^The arrow shows the year of cessation.

In France, the majority (81–92%) of childhood patients with TB were born abroad or had parent(s) from overseas between 2007 and 2018. A change in the number of childhood patients with TB with French-origin parents could not be identified due to non-existent pre-cessation data; however, this number had fluctuated from 20 to 40 after 2007 without any sharp increase, except in 2017 (Fig. 2a, data from Santé Publique France).

Similar to the findings in France, the majority (74–100%) of childhood patients with TB in Norway were born abroad or had parent(s) from overseas from 1999 to 2018, with a mean proportion of 90%, both pre- and post-cessation. The number of children having TB, with Norwegian parents, did not increase after cessation, although these numbers were quite small overall (Fig. 2b, data from the Norwegian Institute of Public Health).

In the UK, the number of UK-born and foreign-born childhood patients with TB showed similar trends since 2000, with both showing a peak in 2008. About 60% of childhood TB patients were UK-born between 2000 and 2013, and this proportion increased to 70% after 2014. There was no obvious change in this percentage around the year of vaccination policy change (Fig. 2c, data from Public Health England). However, the birthplace analysis for the UK was not simple because the influence of UK-born children with foreign-born parents is relatively large (76% of UK-born childhood patients with TB from 2000–2015 were from non-white ethnic groups)^12^.

### Trends in TB notifications based on TB disease type among children with TB (0–14 years of age)

**Figure 3:**
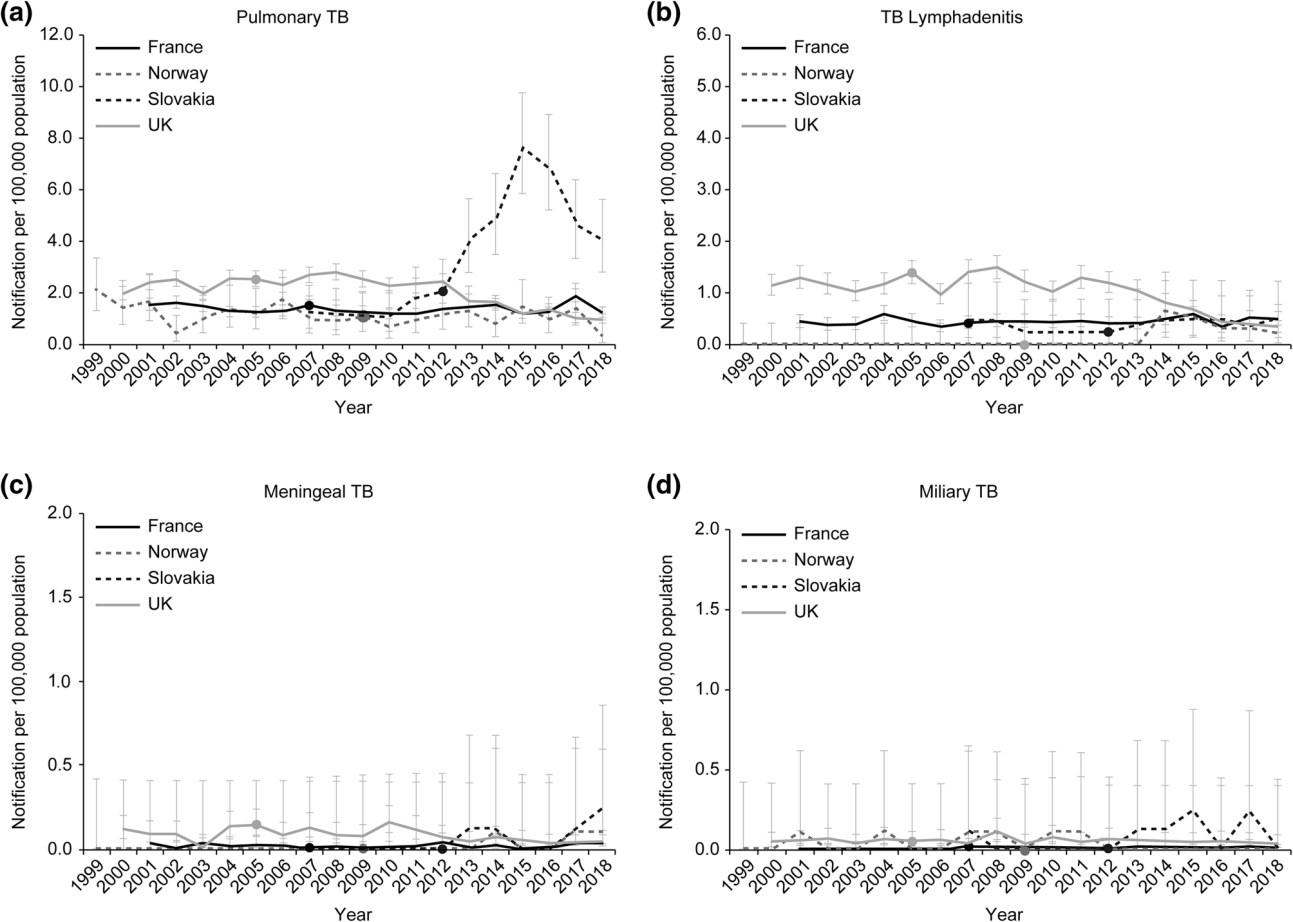
Trends in childhood tuberculosis notification rate based on disease type (0–14 years of age). *Data sources: The World Bank for the population data, Santé Publique France, The Norwegian Institute of Public Health, National Institute for TB, Lung Diseases and Thoracic Surgery in Slovakia, and Public Health England for the number of TB notification. ^†^TB notification rate per 100,000 population; and the dot presents the year of cessation. ^‡^For years of zero notifications of TB lymphadenitis, meningeal TB, and miliary TB, the one-sided confidence interval (97.5%) was used. ^§^In Norway, seven individuals who had multiple infections (pulmonary TB plus lymphadenitis or meningitis) were counted in both organs. There were no cases categorised as TB lymphadenitis before 2013.

In four countries (France, Norway, Slovakia, and the UK), the majority of childhood patients with TB (0–14 years of age) for five years before and after cessation had pulmonary TB and/or TB lymphadenitis (58–100% of total childhood TB cases pre-cessation and 65–96% post-cessation). Severe forms of TB (meningeal and miliary TB) constituted less than 4% of the total childhood TB cases across these periods in these four countries. For the notification rate of pulmonary TB, Slovakia showed a slight increase since 2010 (before the BCG policy change) and a sharp increase for three years after cessation, while other countries had no change (Fig. 3a). For TB lymphadenitis, Slovakia showed a slight increase in cases, while the notification rate in Norway increased in 2014 (five years after cessation) and then slowly decreased (Fig. 3b). Although the number of cases of severe forms of TB (meningeal and miliary TB) was limited, Slovakia showed a slight increase in miliary TB cases (Fig. 3c,d, Table 3, data from the National Institute for TB, Lung Diseases and Thoracic Surgery in Slovakia, Santé Publique France, Norwegian Institute of Public Health, Public Health England)^5^.

Published reports from Sweden also demonstrated that the number of pulmonary TB and TB lymphadenitis cases increased for six years after cessation, while those of meningeal and miliary TB showed a minimal increase^10,13^.

### Vaccine coverage under a selective vaccination program

Most of the 16 countries have administered selective vaccination after cessation, including defining high-risk groups, and two countries (France and the UK) have offered BCG vaccination to neonates and children living in particular areas inside their countries (see Supplementary Table S1)^3,14–27^.

In the UK, the BCG vaccine coverage estimate has been available from 2015 after cessation of universal BCG vaccination, and the proportions of universal BCG coverage were 32–91% in 10 local authorities (LAs) with high TB notification rates (more than 40 per 100,000 populations) where universal BCG vaccination was implemented^28^. In 2018, there were five LAs in London with high TB notification rates and BCG vaccine coverage there ranged from 37 to 69%; although these percentages were low, they were higher than those in 2017 in three LAs^29^. In France, BCG uptake had already dropped one year before cessation due to the change in the vaccination method to an intradermal injection. After cessation, vaccine coverage was made available in eight districts of Ile de France where TB notification rates (all forms, all ages) were 11–24 per 100,000 population (2018). The vaccine coverage there was low (45–68%) for a few years after cessation, but it has recently increased to 73–90% (data from Santé Publique France)^30^.

Vaccine coverage for the target group in the Czech Republic, Norway and Slovenia were 50–80%, 82–86% (2007–2010), and 70–90%, respectively, after shifting to selective vaccination (data from the Institute of Health Information and Statistics of the Czech Republic)^31–33^. BCG uptake in Sweden also dropped after cessation; however, it increased slowly after revision of the recommendations and clarification of the risk group definition. Subsequently, TB notification rates were reduced in the second generation of immigrants^6^.

## Discussion

Among countries where data were available, Slovakia was the only country with an increase in childhood TB notification after cessation of universal BCG vaccination; pulmonary TB and TB lymphadenitis were the major cause of increased childhood TB rates, while changes in the rates of meningeal and miliary TB were small. This is a relatively preferable result for healthcare staff managing childhood patients with TB in the clinical setting. However, maintenance of adequate knowledge and skill for appropriate diagnosis of childhood TB is essential, as the number of patients with TB increases as a result of vaccination cessation. There was an interesting observation that Eastern European countries, namely the Czech Republic, Slovakia and Slovenia, showed different trends in childhood TB after the vaccination policy change. Only Slovakia showed a rapid increase in TB notification, and the majority of this increase was due to cases from the Roma community that was the highest risk population in the country. The causes of this increase might be a combination of a natural increase after terminating universal BCG vaccination, and strengthened surveillance and case detection system that might bring a difference with other countries.

The low BCG vaccine coverage in the target group after shifting to selective vaccination was a common challenge in the Czech Republic, France, Norway, Slovenia, Sweden, and the UK. A study from Sweden showed that TB notification decreased after improving BCG vaccine coverage in the high-risk group^6^. These findings suggest that efforts to maintain a high BCG uptake among high-risk groups are essential and special attention is required at the time of vaccination policy change.

Childhood TB notification rates in six countries (the Czech Republic, Finland, France, Norway, Slovenia, and the UK) that changed their BCG vaccination policy in the 2000s did not increase after cessation of universal BCG vaccination. Four of these countries have had a high proportion of international migrants in the 2000s and had updated their immigration policy in the same period (Table 4)^34–48^. For example, the French immigration and integration law adopted in 2006 promoted selective immigration, which encouraged highly skilled workers and limited family migrants. Thus, net migration (i.e., the difference between the numbers of immigrants and emigrants) rapidly reduced after 2007 (the year of cessation), and the number of family migrants sharply decreased (this number slowly increased again after 2009). The trend in the number of foreign-born childhood patients with TB appeared to be similar to that of the net migration; this is understandable, since childhood TB notifications in France have been driven by foreign-born children and children with parents from abroad, especially those who have recently arrived^34–40^. In the UK, net migration increased in the 2000s as a result of immigration from the newly joined EU countries; however, migration from non-EU citizens has declined steadily since 2005 (the year of cessation), when the government announced a new strategy of immigration control with a point-based system centred on qualifications. Specifically, for TB control, the new pre-entry screening system was implemented in 2012, which was the year that childhood TB notification rates started to decline^44–48^.

**Table 4:** International migrant stock among countries that ceased universal BCG vaccination in the 2000s.

	% of international migrant stock^1^	Year of BCG policy change	Key years regarding immigration policy changes in the 2000s to restrict migration
The Czech Republic	3.9 (2010)	2010	–
Finland	3.7 (2005)	2006	–
France	11 (2005)	2007	2006, 2007^2^
Norway	7.8 (2005)	2009	An immigration act was adopted in 2008 and entered into force in 2010^3^
Slovakia	2.7 (2010)	2012	–
Slovenia	9.9 (2005)	2005	2004, 2008, 2011^4^
The UK	9.8 (2005)	2005	5-year strategy was announced in 2005 ^5^

^1^International migrant stock. World Bank. (https://data.worldbank.org/indicator/SM.POP.TOTL). The data were available every 5 years, and the latest year before the BCG policy change in each country was chosen.

^2^Chou, M. H. & Baygert, N. The 2006 French Immigration and Integration Law: Europeanisation or Nicolas Sarkozy’s presidential keystone? *ESCR Centre on Migration, Policy and Society Working Paper No. 45* https://www.compas.ox.ac.uk/wp-content/uploads/WP-2007-045-Meng-Hsuan-Baygert_French_Immigration_Law.pdf (2007). Kofman, E., Rogoz, M. & Lévy, F. Family migration policies in France. *International Centre for Migration Policy Development: NODE Policy Report* (2010).

^3^International migration 2011–2012*. Norwegian Ministry of Labour* https://www.regjeringen.no/en/dokumenter/international-migration-2011-2012/id711645/ (2013).

^4^Milohnić, A. An overview of the migration policies and trends—Slovenia. Migrationonline.cz.

https://migrationonline.cz/en/e-library/an-overview-of-the-migration-policies-and-trends-slovenia (2013).

^5^United Kingdom Home Office. Controlling our borders: making migration work for Britain—five year strategy for asylum and immigration 1–44. (The Stationery Office, 2005).

For countries where the majority of childhood TB notifications were from foreign-born patients and/or those with parents from abroad, simultaneous sociodemographic changes in the immigrant populations occurred as a result of new immigration measures to restrict the number and types of immigrants; this might have affected childhood TB epidemiology, blurring the effect of BCG vaccination policy change.

The major limitation and challenge of this study was data collection. Systematic data collection was not possible and some data did not exist in country-specific databases. Particularly for birthplace data, the data we could collect were not sufficient to analyse a correlation with vaccination policy change. These limitations in acquiring data made our analysis weaker and our conclusions are supported only by these limited data. Another limitation was the lack of analysis of long-term changes after the vaccination policy change. We aimed to analyse short-term changes since some countries had only recently changed their policy and no long-term data existed. It is possible that the potential long-term impact of vaccination policy change on childhood TB epidemiology might be different to that suggested by our analysis.

Although only a few countries’ examples are provided here, a large amount of heterogeneity in childhood TB epidemiology was seen among these countries. This complexity might occur because TB epidemiology is influenced by several factors such as improvements in case finding and surveillance system, a change of migration population from TB HBCs, as well as the vaccination policy change. Therefore, it is important to monitor and evaluate TB epidemiology in conjunction with these factors, especially regarding the origin country of patients in countries where TB notifications are driven by migrants. These indicators may help define potential groups at a high risk of TB infection in each country. Identification of such groups is key for efficient TB control, in terms of early case finding and maintaining high BCG vaccine uptake under the selective vaccination program. This would be especially important for healthcare staff in low TB burden countries who are not familiar with TB care. However, careful attention is required to prevent discrimination against these high-risk groups—advocating TB and BCG vaccination in both medical and non-medical populations would help raise awareness and reduce stigmatisation.

## Supplementary Information

Supplementary Information.

## Data Availability

The data that support the findings of this study are available from the National TB Surveillance Unit, the Czech Republic; Santé Publique France; Norwegian Institute of Public Health; National Institute for TB, Lung Diseases and Thoracic Surgery, Slovakia; the Swiss Federal Office of Public Health; and Public Health England, the UK, but restrictions apply to the availability of these data, which were used under license for the current study and, therefore, only selected data are publicly available. Data are, however, available from the authors upon reasonable request and with permission from the National TB Surveillance Unit, the Czech Republic; Santé Publique France; Norwegian Institute of Public Health; National Institute for TB, Lung Diseases and Thoracic Surgery, Slovakia; the Swiss Federal Office of Public Health; and Public Health England, the UK.
