# Supplementary material for: Epidemiology of childhood tuberculosis after ceasing universal Bacillus Calmette–Guérin vaccination
Source: Sci Rep. 2021 Aug 5;11:15902. doi: 10.1038/s41598-021-95294-y (PMC8342465; doi:10.1038/s41598-021-95294-y)
Supplement: Supplementary file 1 — Supplementary Information. [file 41598_2021_95294_MOESM1_ESM.docx]

**Epidemiology of childhood tuberculosis after ceasing universal Bacillus Calmette–Guérin vaccination**

Sayori Kobayashi^*^ MD, MPH, Takashi Yoshiyama MD, Kazuhiro Uchimura MSc, Yuko Hamaguchi MPH, MBA, PhD, Seiya Kato MD, PhD

**Supplementary Table S1: Target group for selective vaccination**

| **Country** | **Target group of selective vaccination** |
| --- | --- |
| Australia | 1. Aboriginal and Torres Strait Islander neonates in communities with high TB notification rates 2. Neonates and children <5 years of age who will travel or live in high TB burden countries for extended periods 3. Neonates born to parents with leprosy or with a family history of leprosy   (BCG vaccination may be considered in some occupational groups such as healthcare workers at high risk of exposure to TB, embalmers) |
| Austria | BCG vaccine is no longer recommended |
| Czech Republic | Infants and children:   1. who originate from countries with TB incidence ≥40 per 100,000 or who are/were continuously staying for >3 months in those countries 2. whose sibling(s), parent(s), or member(s) of common household originate from countries with TB incidence ≥40 per 100,000 or are/were continuously staying for >3 months in those countries 3. who have parent(s), sibling(s), or member(s) of common household with a current or history of TB 4. who were in contact with TB |
| Denmark | 1. Newborns whose parents are from TB endemic areas (TB incidence >40 per 100,000) 2. Immigrant children <12 years of age who visit relatives in TB endemic areas 3. Children <12 years of age who stay for a longer period (>6 months) in TB endemic areas 4. Adults at risk of being infected with particularly resistant TB |
| Finland | Newborns and children <7 years of age:   1. who live with a family member/person with a history of TB 2. who were born in a high TB burden country or live with a family member/person who was born in a high TB burden country 3. who will move to a high TB burden country within a year and will stay there more than 1 month |
| France | Children <6 years of age:   1. who were born in a high TB burden country 2. with at least one parent from a high TB burden country 3. who will stay at least 1 month in a high TB burden country 4. whose families have a history of TB 5. who live in Ile de France, Guyane, or Mayotte 6. who are in any situation at risk judged by a doctor |
| Germany | BCG vaccine is no longer available |
| Israel | Immigrant children born in high TB burden countries |
| New Zealand | Babies or children:   1. who will live in a house with a person with either current TB or a history of TB 2. who have one or both parents, household members, or caregivers who lived for ≧6 months in a high TB burden country (≥ 40 per 100,000) within the last 5 years 3. who will live for ≥3 months in a high TB burden country during their first 5 years |
| Norway | 1. Infants with parents from highly endemic countries 2. Previously unvaccinated healthcare personnel and students <35 years of age whose work involves care for adult patients with pulmonary TB in hospitals or cultures of mycobacteria in laboratories |
| Slovakia | Roma ethnic group |
| Slovenia | Newborns and infants of immigrant families who moved to Slovenia from high TB burden countries within the last 5 years |
| Spain | 1. Infants and children who were in contact with TB patients 2. Immigrant children <5 years of age from high TB burden countries (≥40 per 100,000) who will return to their country of origin to stay >3 months or are expected to travel to the country repeatedly during childhood 3. Children <5 years of age of workers who come from these countries, who will travel and/or live with the local population for >3 months in these countries 4. Healthcare workers frequently in contact with TB patients or laboratory samples |
| Sweden | 1. Children up to 6 years of age with a family origin from a country with increased or high TB burden   Children and adolescents up to 18 years of age:  1. in contact with a current TB patient (close relative or household member)  2. who stay >3 months in high TB burden countries and are in close contact with the local population |
| Switzerland | Newborns and infants <12 months of age:   1. whose parents originate from high TB burden countries (>50 cases per 100,000 per year) 2. who will return to these countries |
| United Kingdom | All babies ≦1 year of age:   1. who were born in areas of the UK with TB notification rates of ≥40 per 100,000 2. whose parent(s) or grandparent(s) were born in a high TB burden country   All older children and adults at risk of TB, including:   1. older children with an increased risk of TB who were not vaccinated against TB when they were babies 2. anyone <16 years of age who has come from a high TB burden country 3. anyone <16 years of age who has been in close contact with pulmonary TB patients, or who will live with local people for >3 months in an area of high TB burden 4. adults ≦35 years of age at occupational risk of TB infection (staff who work in a laboratory, veterinary clinic, prison, hostel for the homeless, refugees, and asylum seekers, and other healthcare workers at risk of TB exposure) |

Abbreviations: Bacillus Calmette–Guérin (BCG); tuberculosis (TB);
